# Successful Treatment of Human Monocytic Ehrlichiosis with Rifampin

**DOI:** 10.7759/cureus.444

**Published:** 2016-01-01

**Authors:** Khalid Abusaada, Saira Ajmal, Laura Hughes

**Affiliations:** 1 Graduate Medical Education, Florida Hospital-Orlando; 2 Infectious Diseases, Mayo Clinic, Rochester, MN, USA

**Keywords:** ehrlichia, human monocytic ehrlichiosis, rifampin, therapy

## Abstract

Currently recommended treatment regimens for human monocytic ehrlichiosis (HME) include doxycycline or tetracycline. Antibiotic susceptibility studies demonstrate that rifampin has in vitro bactericidal activity against Ehrlichia. Case reports have suggested clinical response with rifampin treatment of human granulocytic anaplasmosis (HGA). We report the first case of HME successfully treated with rifampin.

## Introduction

Human monocytic ehrlichiosis (HME) is a tickborne zoonotic infection caused by the obligate intracellular bacterium, *Ehrlichia chaffeensis,* that infects monocytes [1]. Ehrlichiosis was initially recognized as a human disease in the 1980s [2]. According to data collected by the Center for Disease Control (CDC), most reported cases are located in the southeastern and south-central United States, corresponding to the known distribution of the lone star tick (*Amblyomma americanum*), which has been implicated in the transmission of the bacteria [2]. According to a 2006 report from the CDC, the average reported annual incidence of HME was 0.7 cases per million population, based on data from 2001-2002; however, it was noted that this is likely an underestimation of the true incidence in endemic areas [1].

Current recommendations advocate the use of doxycycline as the preferred drug for treatment [3]. This recommendation is largely based on empirical data and clinical experience, as no clinical trials have been conducted [3]. There are few data available supporting alternative regimens for situations in which doxycycline is contraindicated [3]. Cases in the medical literature report the successful treatment of human granulocytic anaplasmosis (HGA) with rifampin in pregnant females and children [4-5]. We report a case of a non-pregnant female with acute HME who was treated successfully with rifampin.

## Case presentation

A 64-year-old female from central Florida presented with generalized fatigue, malaise, myalgias, and arthralgias for four days. She also reported subjective fevers and a nonproductive cough for one day. She had recently gone hiking in central Florida where she was bitten by several ticks two weeks prior to presentation. She reported identifying and removing six ticks that were attached to her legs the day after her hiking trip. Two of the ticks were engorged.

Physical examination was unremarkable, apart from tender lymphadenopathy of the right anterior cervical chain. Informed patient consent was obtained for treatment.

Her white blood cell count (WBC) was 3130 /µl, with 87% neutrophils and 7.3% lymphocytes. Her hematocrit was 44.3%, and her platelet count was 144,000 /µl. Liver function tests showed a normal bilirubin of 0.3 mg/dl (normal 0.1-1.5 mg/dl) and alkaline phosphatase level of 97 units/dl (normal 14-127 units/dl). AST and ALT were both elevated with an AST of 67 units/dl (normal 5-46 units/dl) and an ALT of 63 units/dl (normal 4-51 units/dl). Influenza A and B were negative by polymerase chain reaction (PCR) testing of a nasopharyngeal swab. Her chest x-ray was normal. 

On the second day of hospitalization, her WBC count decreased further to 1870/µl, with 60% bands, 16% neutrophils, 18% lymphocytes, and the patient's platelet count dropped to 91,00/µl. HME or HGA was suspected and, due to a history of doxycycline allergy, the patient was started on oral rifampin, 300 mg twice daily.

*Ehrlichia chaffeensis* serology returned negative (IgM < 1:16, IgG < 1:64). Polymerase chain reaction (PCR) analysis of the patient’s blood for *A. phagocytophilum* was negative; however, the 16S rRNA gene of *E. chaffeensis* was detected by PCR. 

The patient was continued on rifampin therapy for HME. She improved clinically, both by symptomatology as well as laboratory findings, and was discharged on hospital day 6, to complete a seven-day course of rifampin.

At follow-up three weeks later, she had no symptoms. Her CBC, platelet count, and hepatic transaminases were normal.

## Discussion

Human ehrlichiosis (HE) is a serious illness. Physicians in endemic areas should have a high index of suspicion for HE and initiate empiric antimicrobial therapy when appropriate [1, 6]. It is reported that delaying treatment may also lead to a more severe disease course and possibly fatal outcomes [1]. Case fatality for HME is about 3% [3]. Life-threatening complications, such as septic shock-like syndrome, hemorrhage, acute renal failure, myocarditis, and meningoencephalitis, develop in about 17% of HME patients [3]. A case report from Texas suggests that *E. chaffeensis* can cause persistent infection in humans, further emphasizing the importance of early treatment [7].

Typically, patients present with nonspecific symptoms, including fever, myalgia, arthralgia, fatigue, headache, nausea, and vomiting. Typical laboratory findings include elevated AST and ALT, lymphopenia, and thrombocytopenia [3, 8-10]. The microbiological diagnosis of ehrlichiosis is challenging due to the characteristics of the organism. As an obligate intracellular bacteria, *Ehrlichia* requires a suitable continuous cell line for growth [11]. Furthermore, serologies may remain negative for up to three weeks after symptom onset [9]. The gold standard serologic test is a four-fold increase in the IgG-specific antibody titers using immunofluorescence (IFA) on paired samples from week 1 and weeks 2-4 of the illness [10]. Microscopic examination of Wright-stained peripheral blood smears may demonstrate morulae (intracytoplasmic inclusions) during acute illness; however, this is a relatively insensitive test for HME with < 10% of patients displaying this finding [3]. PCR amplification of specific DNA has been found to be a helpful method for diagnosis in the acute phase of ehrlichiosis and is becoming the diagnostic test of choice [3, 9].

Clinical data on the treatment of ehrlichiosis is limited [12]. Historically, doxycycline and tetracycline have been recommended as first-line therapy for HME, with chloramphenicol as a possible alternative [6, 9]. However, evaluation of antibiotic susceptibilities of *Ehrlichia* species reveal that chloramphenicol is ineffective in vitro against *E. sennetsu*, *E. canis*, and *E. chaffeens*is [8], which discourages its use in the treatment of HME and HGA [3]. Currently, recommended regimens include doxycycline, 100 mg orally or intravenously twice daily, or tetracycline, 500 mg orally every six hours, both for a duration of five to 14 days with at least three to five days of antibiotics after the fever subsides [3, 6]. Alternative recommendations for treatment of ehrlichiosis are needed for specific patient populations in whom doxycycline is contraindicated, i.e. pregnant females and patients with allergies or gastric intolerance [12]. Antibiotic susceptibility studies show that doxycycline and rifampin have high in vitro bactericidal activity against *Ehrlichia chaffeensis* [8, 12]. Because the available medical literature on rifampin showed in vitro susceptibility with low minimum inhibitory concentrations (MIC) [3], and case reports indicated successful treatment of HGA in children and pregnant women, we decided to treat our patient with rifampin. We used the regimen reported in literature: 300 mg orally twice daily for seven to ten days, with three to five days of antibiotics after defervescence [3]. Our patient responded to this treatment. Although further studies are needed, in our single case experience, rifampin proved to be an appropriate alternative to doxycycline in the treatment of HME.

## Conclusions

Physicians in endemic areas should have a high index of suspicion for human ehrlichiosis infection and should initiate empiric antimicrobial therapy when appropriate. We present a case of HME resolution following treatment with rifampin in a patient with doxycycline allergy. The treament was well tolerated with rapid resolution of symptoms and correction of laboratory abnormalilties. More research is needed to evaluate clinical effectiveness of rifampin in treament of HME.
